# Change of serum lipoproteins and its potential use in stratifying patients with sepsis among neonates

**DOI:** 10.1186/s40001-023-01077-8

**Published:** 2023-03-01

**Authors:** Junfei Guo, Weiming Lai, Yongbing Wu, Huan Li, Zhenhua Fu, Xiaoping Mu

**Affiliations:** Clinical Laboratory Department, Guangdong Women and Children Hospital, Guangzhou, 511400 China

**Keywords:** Lipoproteins, Bacteremia, Sepsis, HDL-C, LDL-C

## Abstract

**Background:**

Changes of serum lipoprotein concentration during bacteremia or sepsis are observed and lipoproteins concentration facilitate the evaluation severity of sepsis in adults, but its clinical usage is still unclear. Here, we analyzed the lipoprotein concentration in neonates with sepsis and discussed its use in stratifying patients.

**Methods:**

This is a retrospective study involved 88 culture-proven septic patients. Clinical and microbiology data of involved patients were collected via inquiring databases of our institute. Patients were grouped according to blood culture results or procalcitonin level; the difference between groups were analyzed.

**Results:**

Compared with uninfected group, there is no change of triglyceride (TG) concentrations and significant decrease of Total cholesterol (TC) concentration in septic patients. There is no significant difference between Gram-positive and Gram-negative-related septic patients in terms of serum TG and TC concentration. Other than group with procalcitonin level of 0.5–2 ng/ml, both serum TG and TC concentration were decreased while serum procalcitonin level increasing.

**Conclusions:**

Our results indicated that serum lipoprotein concentration may be recommended to help diagnosis of bacteria and to evaluate the severity of sepsis.

## Background

Although significant advances have been made in dealing with bacteremia, it remains an intractable condition with high morbidity and mortality rates in clinical practice worldwide [1]. Prompt identification of bacteremia and timely antimicrobial treatment are pivotal in reducing sepsis-related deaths [2, 3]. Blood culture is the golden standard for the diagnosis of bacteremia, but time consumption and false negatives weaken its clinical usage, especially in the neonatal population. Thus, the development of new biomarkers to facilitate the early identification of bacteremia or the initiation of microbiological examination is of great importance. Several recent studies have demonstrated that serum lipoprotein concentrations were changed during sepsis in adults, and lipoprotein concentration can be used as a prognostic factor for severe sepsis [4, 5], but its usage among neonates population is insufficient study.

Precisely identify the severity of septic patient is important in management of patients. PCT is considered a good indicator to evaluate the severity of sepsis, but its serum concentration may be affected by situations, such as trauma [6], malignancy diseases [7] and physiological conditions of patients [8, 9]. Physiological elevation of PCT among neonates further weaken its usage among neonatal patients. Whether serum lipoprotein concentration can be used to stratify patient with sepsis remains to be tested.

Here, we collected and analyzed the data of serum lipoproteins in neonates with sepsis at our institute. Serum lipoprotein concentration was significantly decreased in neonates with sepsis, and no significant difference was observed between septic patients infected with Gram-positive and Gram-negative bacteria. In PCT-stratified patients, serum lipoprotein concentration gradually decreased, while PCT level increased, indicating the potential use of serum lipid levels in stratifying septic patients. Our study as well as other related studies imply the involvement of serum lipoproteins in neonatal septic patients and it may be use as an indicator to evaluated the severity of sepsis among neonates.

## Methods

### Study population

This is a retrospective cohort study carried out at Guangdong Women and Children Hospital, a 1500-bed tertiary women and children health care center located in southern of China. The study was proved by the Ethics Committee of our institute.

The study population included inpatients who had simultaneous have blood cultures and serum lipoprotein results from Jan. 2015 to Dec. 2019. In our study, only those with serum lipoprotein results within 24 h before or after blood culture positive were included. Patients with known diseases or factors leading to abnormal serum lipoprotein levels or malnutrition (low albumin level) were excluded. In total, 88 neonates meeting the criteria were included and the clinical and laboratory records of these patients were reviewed. A cohort of matched healthy individuals with serum lipoprotein results was also involved, to serve as a control group.

### Data collection

The clinical records of the involved patients were review for the following information: Demographic characteristics, diseases information. The results of laboratory test were acquired via reviewing our laboratory data base. Results of blood culture, serum lipoprotein results, blood routine examination results, C-response protein (CRP) and amyloid A results, procalcitonin (PCT) results, albumin results as well as ferritin results were collected. Here, we only collected the data within 24 h ahead or after the time of blood culture.

### Data analyses and statistics

We first separated the involved patients according to results of blood culture; the data of TG, TCH, HDL-C and LDL-C of each group were calculated and compared. Student’s t test was conducted to evaluated the difference between groups. To further evaluate the change of serum lipids during bacteremia, we stratified the patients according to the results of PCT test: PCT < 0.5 ng/ml, PCT 0.5–2 ng/ml, PCT 2–10 ng/ml and PCT > 10 ng/ml. Serum lipids, white blood cell count, platelet count, neutrophil-to-lymphocyte ratio (NLR) and CRP level of each group were calculated and Student’s *t* test was conducted to evaluated the difference between groups. A *p* ≤ 0.05 was considered statistical difference.

## Results

Table 1 summarizes the demographic characteristics and pathogen composition of the study participants. In all 88 neonates were involved. Gram-negative bacillus were the mainly isolated pathogens, and *E.*coli and *K.* pneumoniae represented 36% and 11% of all isolated pathogens, respectively.

**Table 1:** Demographic characteristics and the pathogen composition of involved patients

Items	Number	Percentage (%)
Gender (male)	47	53
Pathogens		
*E.*Coli	23	26
*K*.Pneumoniae	10	11
Other G- bacteria	10	11
G + bacteria	30	34
Fungus	7	8

**Fig. 1:**
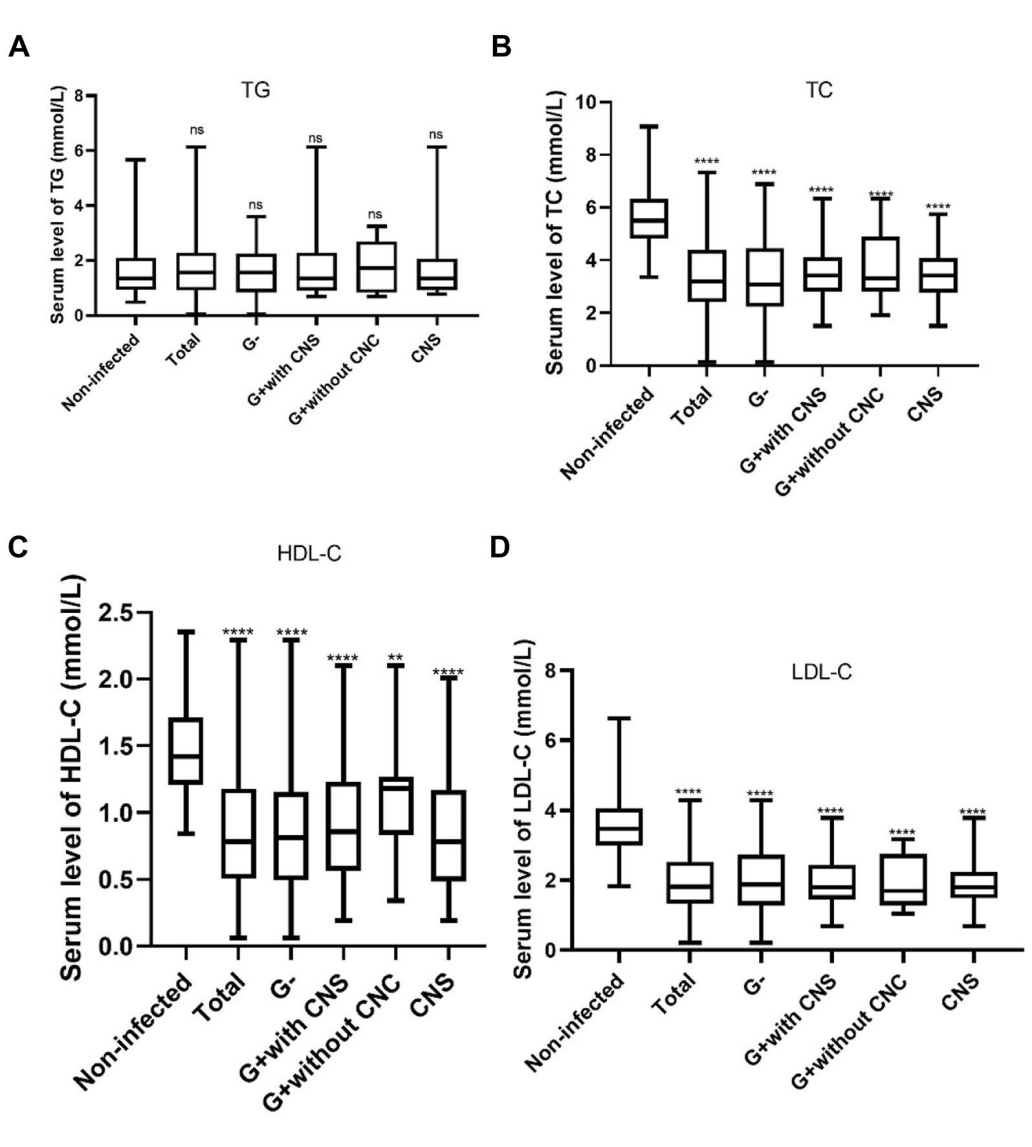
Lipoproteins concentration of patients with bacteremia caused by different pathogens. Groups were compared using Student’s *t* test. **p value ≤ 0.01, ****p value ≤ 0.0001. ns not significant, HDL-C high-density lipoprotein cholesterol, LDL-C low-density lipoprotein cholesterol, TG triglycerides, TC total cholesterol, G- Gram-negative bacterium, G + Gram-positive bacterium, CNS coagulase negative staphylococcus

There was no significant change in TG concentrations in the bacteremia group compared with healthy controls, and no statistically significant differences were found among neonates infected with different pathogens (Fig. 1A). Total cholesterol (TC) of neonates with bacteremia was significantly reduced compared with healthy controls, and neonates with G^−^negative bacteremia had the lowest TC level but no statistically significant differences were observed (Fig. 1B). In accordance with TC distribution, HDL-C and LDL-C concentrations were significantly reduced in neonates with bacteremia (Fig. 1C, D). Although no statistical differences were observed, the G^+^ without CNS group had the highest HDL-C level and lowest LDL-C level (Fig. 1C, D).

**Fig. 2:**
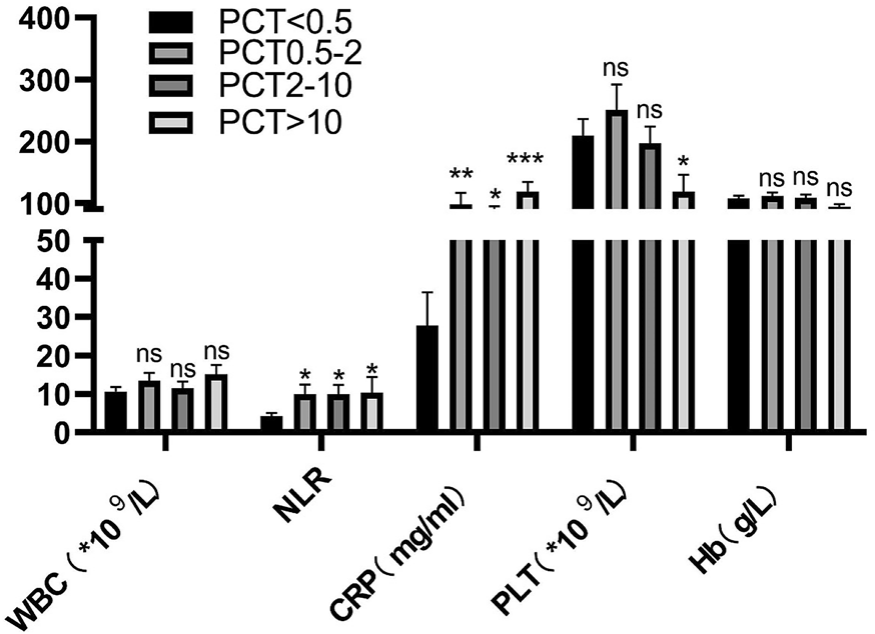
Inflammatory parameters in patients with different PCT levels. Groups were compared using Student’s *t* test. *p value ≤ 0.05, ns not significant, WBC white blood cell, NLR neutrophil-to-lymphocyte ratio, CRP C-responsive protein, PLT platelet, Hb hemoglobin

**Fig. 3:**
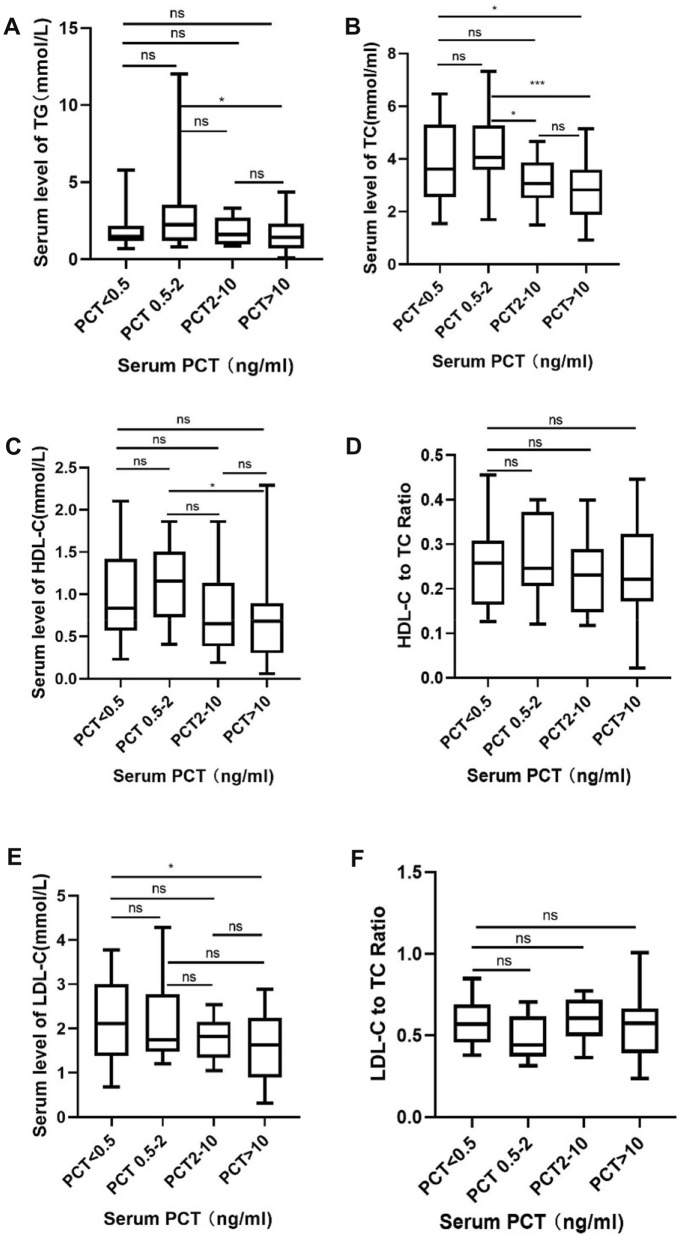
Lipoproteins concentration of patients with different PCT levels. Groups were compared using Student’s *t* test. *p value ≤ 0.05, ***p value ≤ 0.001, ns not significant, HDL-C high-density lipoprotein cholesterol, LDL-C low-density lipoprotein cholesterol, TG triglycerides, TC total cholesterol, PCT procalcitonin

To further reveal the changes in serum lipoproteins during bacteremia and to determine whether lipoprotein concentration correlates well with the severity of disease, we stratified the patients according to serum PCT level, which performs well in recognizing the severity of bacteremia [10]. As shown in Fig. 2, NLR and serum CRP levels correlated well with serum PCT levels in stratified patients, and platelet count was reduced, while PCT level increased (Fig. 2). White blood cell counts and Hb concentrations were not significantly different among PCT-stratified patients (Fig. 2). In general, serum TG and TC concentrations decreased with the increase of PCT level, but there was an increase in TG or TC concentration in the PCT 0.5–2 ng/ml group (Fig. 3A, B). Serum HDL-C concentration decreased, while serum PCT level increased, and similar to TG and TC, there was an increase in HDL-C concentration in the PCT 0.5–2 ng/ml group (Fig. 3C). The HDL-C-to-TC ratio (HTR) decreased while serum PCT increasing (Fig. 3D). The serum LDL-C concentration and LDL-C-to-TC ratio (LTR) decreased while the serum PCT level increasing (Fig. 3E, F), and the PCT 0.5–2 ng/ml group had the lowest LDL-C concentration and LTR.

## Discussion

Here, we present our study focused on the change in serum lipoprotein levels in neonatal septic patients. Our results demonstrated a significant decrease of TC, HDL-C and LDL-C in neonates with sepsis as compared with uninfected control. This is in accordance with the results of previous study [11–14], demonstrating a potential role of serum lip in the course of sepsis. In our current study, we found no significant change in serum TG levels in neonates with sepsis compared to healthy controls. Reasons for the different trends in cholesterol-related lipoproteins (TC, HDL-C and LDL-C) and triglyceride in septic patients were unclear. Different affinity of these lipids-to-pathogen-related lipopolysaccharide (LPS) or lipoteichoic acid (LTA) [15, 16] may be part of the reasons. Though no significant differences were found between G-and G + bacteremia patients in terms of serum lipoprotein level, G-bacteremia patients had a lower TG and TC levels; LPS from Gram-negative pathogens influenced chemokine induction more extensively than lipoteichoic acid from Gram-positive bacteria might explain the phenomenon [17, 18].

The clinical manifestations of sepsis caused by different bacteria are similar, but the targeted antibiotic therapies for Gram-positive and Gram-negative bacterial sepsis are different. Other than pathogen isolation, commonly used biomarkers lack the ability to differentiate Gram-positive bacteremia from Gram-negative bacteremia. A previous study by Alex found that patients with Gram-negative bacteremia had higher TG level than patients with Gram-positive bacteremia [19]. We found no difference between Gram-positive and Gram-negative bacteremia in terms of serum lipoprotein concentrations. Different patient inclusion criteria might be one of the reasons, and the relatively small sample size of our study population may introduce study bias.

It is important to precisely identify the severity of septic patients. Many biomarkers, such as CRP, PCT and SAA, could be used in the auxiliary diagnosis of infectious diseases, but their serum levels may be affected by many other stimulations other than infection [6–8]. Few biomarkers could identify the severity of sepsis. Currently, PCT is considered a good marker for stratify septic patients [10, 20]. Here, we found that serum levels of all kinds of lipoprotein concentrations were significantly decreased, while PCT level increased, implying the potential use of lipoprotein concentration in the stratification of septic patients. Several previous studies have demonstrated that serum levels of HDL-C and LDL-C were negatively correlated with the severity of sepsis [11, 12, 14]. Lower HDL-C level correlate with poor prognosis of the septic patients [14, 21]. The potential use of serum lipids in stratifying septic patients has its advantages: serum lipids test is widely carried out in clinical practice. There is an elevation “time-course” of biomarkers such as CRP, PCT and SAA, their serum levels might be different at different time points of the disease [22–24], and thus, it may introduce bias while using them to evaluate the severity of sepsis. A previous study found that serum HDL-C levels were sustained in septic patients during the course of the disease [25]. Several patients with septic shock in our study had sustained low serum lipids levels (data not show). Together, these results imply a potential role of serum lipids level in stratifying septic patients.

We also observed an interesting phenomenon: there was an increase in serum TG, TC and HDL-C concentrations in the group of patients with PCT level between 0.5 and 2 ng/ml. We speculated that this might be a protective mechanism in the body, as both of these lipoproteins are capable of neutralizing pathogen-related LPS or LTA [15, 16]. An increase in lipoprotein concentration in this subgroup of patients may conceal the manifestation caused by LPS or LTA, and this possibility should be considered in clinical practice. Further investigations are required to explain this phenomenon.

The retrospective and single-center-based characteristics of the current study have several limitations. The baseline level of lipoprotein in our study patients was unavailable; thus, it is difficult for us to evaluate the causal relationship between lipoprotein concentration and bacteremia. A multicenter prospective study may better solve these problems.

## Conclusions

Together, our study reveals the change in lipoprotein levels and their potential use in stratifying patients with sepsis among neonates. Serum lipoprotein concentration may be recommended to help diagnose bacteremia and evaluate the severity of sepsis. Further studies are required to confirm this hypothesis.

## Data Availability

All data generated or analysed during this study are included in this published article.

## Abbreviations

TC: Total cholesterol
TG: Triglyceride
LDL-C: Low density cholesterol
HDL-C: High density cholesterol
CRP: C-response protein
PCT: Procalcitonin
LPS: Lipopolysaccharide
LTA: Lipoteichoicacid
